# Crystal structure of di­chlorido­bis­(4-ethyl­aniline-κ*N*)zinc

**DOI:** 10.1107/S2056989014027832

**Published:** 2015-01-14

**Authors:** J. Govindaraj, S. Thirumurugan, D. Snehalatha Reddy, K. Anbalagan, A. SubbiahPandi

**Affiliations:** a Department of Physics, Pachaiyappa’s College for Men, Kanchipuram 631 501, India; b Department of Chemistry, Pondicherry University, Pondicherry 605 014, India; c Department of Physics, Presidency College (Autonomous), Chennai 600 005, India

**Keywords:** crystal structure, zinc complex, tetra­hedral coordination, hydrogen bonding

## Abstract

The title compound, [ZnCl_2_(C_8_H_11_N)_2_], was synthesized by the reaction of zinc dichloride and 4-ethyl­aniline. The Zn^2+^ cation is coordinated by two Cl^−^ anions and the N atoms of two 4-ethyl­aniline ligands, forming a distorted Zn(N_2_Cl_2_) tetra­hedron. The dihedral angle between the two benzene rings is 85.3 (2)° The Zn atom lies on a twofold rotation axis. The ethyl substituents are disordered over two sets of sites in a 0.74 (2):0.26 (2) ratio. In the crystal, N—H⋯Cl hydrogen bonds link the mol­ecules into sheets perpendicular to the *a* axis. C—H⋯Cl inter­actions also occur.

## Related literature   

For the biological activity and potential applications of mixed-ligand di­chlorido­zinc complexes, see: Tang & Shay (2001 ▸); Lynch *et al.* (2001 ▸); Coulston & Dandona (1980 ▸); May & Contoreggi (1982 ▸). For a related structure, see; Ejaz *et al.* (2009 ▸). 
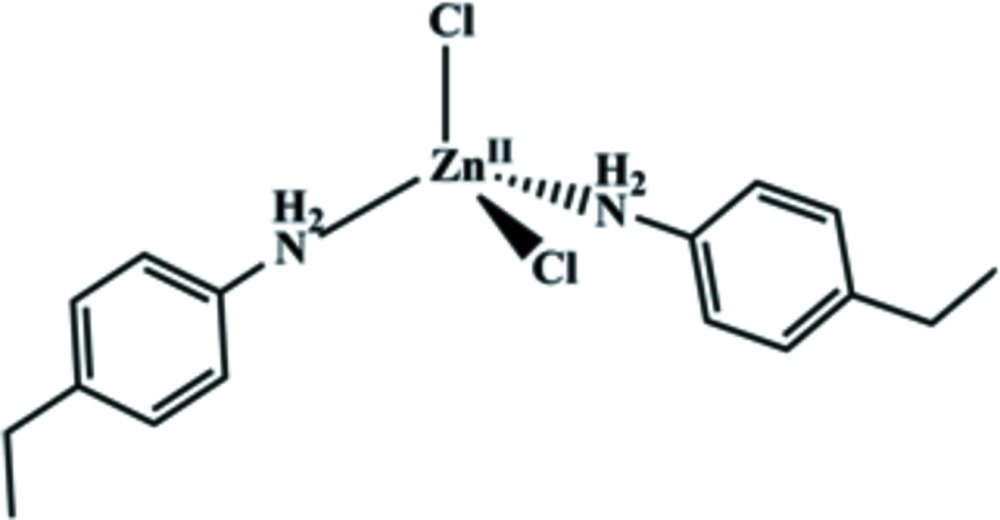


## Experimental   

### Crystal data  

[ZnCl_2_(C_8_H_11_N)_2_]
*M* *_r_* = 378.63
Monoclinic, 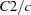

*a* = 32.7291 (16) Å
*b* = 4.7499 (4) Å
*c* = 11.6479 (8) Å
β = 98.016 (7)°
*V* = 1793.1 (2) Å^3^
*Z* = 4
Mo *K*α radiation
μ = 1.66 mm^−1^
*T* = 293 K
0.35 × 0.30 × 0.25 mm

### Data collection  

Oxford diffraction Xcalibur diffractometer with an Eos detector
Absorption correction: multi-scan (*CrysAlis PRO*; Oxford Diffraction, 2009 ▸) *T*_min_ = 0.564, *T*_max_ = 0.660
4578 measured reflections
1578 independent reflections
1440 reflections with *I* > 2σ(*I*)
*R*_int_ = 0.029

### Refinement  

*R*[*F* ^2^ > 2σ(*F* ^2^)] = 0.028
*wR*(*F* ^2^) = 0.077
*S* = 1.10
1578 reflections
123 parameters
66 restraints
H atoms treated by a mixture of independent and constrained refinement
Δρ_max_ = 0.46 e Å^−3^
Δρ_min_ = −0.28 e Å^−3^

### 

Data collection: *CrysAlis CCD* (Oxford Diffraction, 2009 ▸); cell refinement: *CrysAlis RED* (Oxford Diffraction, 2009 ▸); data reduction: *CrysAlis RED*; program(s) used to solve structure: *SHELXS97* (Sheldrick, 2008 ▸); program(s) used to refine structure: *SHELXL97* (Sheldrick, 2008 ▸); molecular graphics: *ORTEP-3 for Windows* (Farrugia, 2012 ▸) and *DIAMOND* (Brandenburg, 2006 ▸); software used to prepare material for publication: *SHELXL97* and *PLATON* (Spek, 2009 ▸).

## Supplementary Material

Crystal structure: contains datablock(s) global, I. DOI: 10.1107/S2056989014027832/ff2133sup1.cif

Structure factors: contains datablock(s) I. DOI: 10.1107/S2056989014027832/ff2133Isup2.hkl

**Fig. 1.**
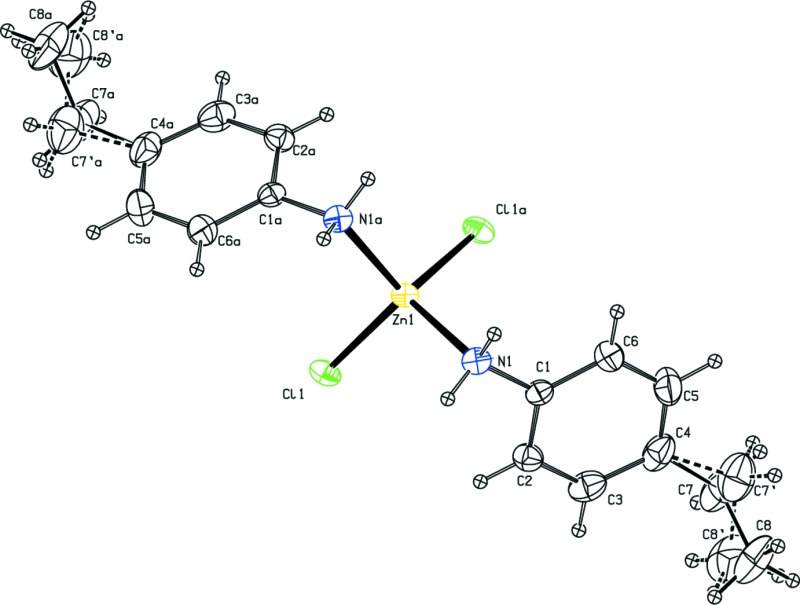
The molecular structure of the title compound with displacement ellipsoids drawn at the 30% probability level.

**Fig. 2.**
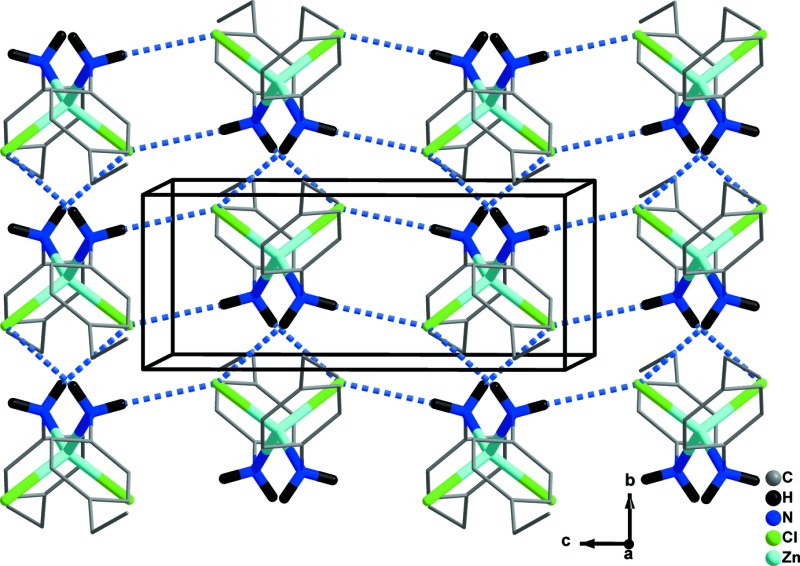
The crystal packing of the title compound viewed down the a axis showing the hydrogen bonded sheet. Hydrogen bond are shown as dashed lines. The minor disorder component and hydrogen atoms not participating in N—H···Cl interactions are omitted for clarity.

. DOI: 10.1107/S2056989014027832/ff2133fig1.tif
The mol­ecular structure of the title compound with displacement ellipsoids drawn at the 30% probability level.

a . DOI: 10.1107/S2056989014027832/ff2133fig2.tif
The crystal packing of the title compound viewed down the *a* axis showing the hydrogen bonded sheet. Hydrogen bond are shown as dashed lines. The minor disorder component and hydrogen atoms not participating in N—H⋯Cl inter­actions are omitted for clarity.

CCDC reference: 1040586

Additional supporting information:  crystallographic information; 3D view; checkCIF report

## Figures and Tables

**Table 1:** Hydrogen-bond geometry (, )

*D*H*A*	*D*H	H*A*	*D* *A*	*D*H*A*
C2H2Cl1^i^	0.93	2.94	3.630(2)	132
N1H1*A*Cl1^ii^	0.88(2)	2.65(2)	3.424(2)	149(2)
N1H1*B*Cl1^iii^	0.88(2)	2.66(2)	3.5083(19)	161(2)

Symmetry codes: (i) 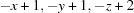; (ii) 
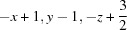
; (iii) 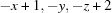.

## supplementary crystallographic information

### S1. Comment

Zinc has many biological functions. It is considered to be an essential nutrient that is required for optimal growth and normal development of vertebrate organisms, as well as being important for maintaining the structure of many proteins. From previous research results, it has been known for many years that zinc mimics the actions of insulin on cells, including promotion of both lipogenesis and glucose transport. Zinc deficiency may indeed affect the optimal functioning of the insulin-signaling pathway (Tang & Shay, 2001; Lynch *et al.*, 2001; Coulston & Dandona, 1980; May & Contoreggi, 1982).

In the title compound (I), (Fig. 1), the Zn^2+^ cation lies on a crystallographic twofold rotation axis, with one half of the molecule connected to the other on by this symmetry operation. The bond distance Zn—Cl and Zn—N are 2.2409 (6) and 2.048 (2) Å, and the bond angles Cl—Zn—Cl and N—Zn—N are 109.41 (3) and 114.80 (11)°. All bond lengths and bond angles in (I) are in the range of expected values. The dihedral angle between the aromatic rings of the 4-ethylaniline ligands is 85.3 (2)°.

N—H···Cl hydrogen bonds serve to link the molecules into sheets perpendicular to the *a* axis.

### S2. Experimental

The title compound was synthesized using zinc chloride (0.5 g, 1 mmol) and 4-ethylaniline (0.91 ml, 2 mmol) in 20 ml of ethanol stirring for 2 h. Colorless crystals were obtained and recrystallized from ethanol. The resulting solution was subjected to crystallization by slow evaporation of the solvent resulting in single crystals suitable for X-ray crystallographic studies.

### S3. Refinement

N and C-bound H atoms were positioned geometrically (C–H = 0.93–0.97 Å) and allowed to ride on their parent atoms, with *U*_iso_(H) = 1.5*U*_eq_(C) for methyl H atoms and 1.2*U*_eq_(C) for all other H atoms.

### Crystal data

**Table:**

[ZnCl_2_(C_8_H_11_N)_2_]	*F*(000) = 784
*M**_r_* = 378.63	*D*_x_ = 1.403 Mg m^−^^3^
Monoclinic, *C*2/*c*	Mo *K*α radiation, λ = 0.71073 Å
Hall symbol: -C 2yc	Cell parameters from 5623 reflections
*a* = 32.7291 (16) Å	θ = 2.6–24.9°
*b* = 4.7499 (4) Å	µ = 1.66 mm^−^^1^
*c* = 11.6479 (8) Å	*T* = 293 K
β = 98.016 (7)°	Block, colourless
*V* = 1793.1 (2) Å^3^	0.35 × 0.30 × 0.25 mm
*Z* = 4	

### Data collection

**Table:**

Oxford diffraction Xcalibur diffractometer with an Eos detector	1578 independent reflections
Radiation source: fine-focus sealed tube	1440 reflections with *I* > 2σ(*I*)
Graphite monochromator	*R*_int_ = 0.029
ω and φ scans	θ_max_ = 25.0°, θ_min_ = 4.6°
Absorption correction: multi-scan (*CrysAlis PRO*; Oxford Diffraction, 2009)	*h* = −36→38
*T*_min_ = 0.564, *T*_max_ = 0.660	*k* = −5→5
4578 measured reflections	*l* = −13→13

### Refinement

**Table:**

Refinement on *F*^2^	Primary atom site location: structure-invariant direct methods
Least-squares matrix: full	Secondary atom site location: difference Fourier map
*R*[*F*^2^ > 2σ(*F*^2^)] = 0.028	Hydrogen site location: inferred from neighbouring sites
*wR*(*F*^2^) = 0.077	H atoms treated by a mixture of independent and constrained refinement
*S* = 1.10	*w* = 1/[σ^2^(*F*_o_^2^) + (0.0411*P*)^2^] where *P* = (*F*_o_^2^ + 2*F*_c_^2^)/3
1578 reflections	(Δ/σ)_max_ = 0.001
123 parameters	Δρ_max_ = 0.46 e Å^−^^3^
66 restraints	Δρ_min_ = −0.28 e Å^−^^3^

### Special details

**Table:**

Geometry. All e.s.d.'s (except the e.s.d. in the dihedral angle between two l.s. planes) are estimated using the full covariance matrix. The cell e.s.d.'s are taken into account individually in the estimation of e.s.d.'s in distances, angles and torsion angles; correlations between e.s.d.'s in cell parameters are only used when they are defined by crystal symmetry. An approximate (isotropic) treatment of cell e.s.d.'s is used for estimating e.s.d.'s involving l.s. planes.
Refinement. Refinement of *F*^2^ against ALL reflections. The weighted *R*-factor *wR* and goodness of fit *S* are based on *F*^2^, conventional *R*-factors *R* are based on *F*, with *F* set to zero for negative *F*^2^. The threshold expression of *F*^2^ > σ(*F*^2^) is used only for calculating *R*-factors(gt) *etc*. and is not relevant to the choice of reflections for refinement. *R*-factors based on *F*^2^ are statistically about twice as large as those based on *F*, and *R*- factors based on ALL data will be even larger.

### Fractional atomic coordinates and isotropic or equivalent isotropic displacement parameters (Å^2^)

**Table:**

	*x*	*y*	*z*	*U*_iso_*/*U*_eq_	Occ. (<1)
C1	0.41733 (7)	−0.0083 (5)	0.81147 (18)	0.0354 (5)	
C2	0.41424 (8)	0.1721 (5)	0.90189 (19)	0.0434 (6)	
H2	0.4358	0.1863	0.9628	0.052*	
C3	0.37917 (9)	0.3321 (6)	0.9021 (2)	0.0583 (7)	
H3	0.3775	0.4538	0.9638	0.070*	
C4	0.34669 (9)	0.3176 (7)	0.8144 (3)	0.0638 (8)	
C5	0.35114 (10)	0.1405 (8)	0.7234 (3)	0.0727 (9)	
H5	0.3300	0.1306	0.6613	0.087*	
C6	0.38593 (9)	−0.0229 (6)	0.7211 (2)	0.0557 (7)	
H6	0.3880	−0.1417	0.6586	0.067*	
C7	0.3096 (3)	0.505 (3)	0.8230 (13)	0.096 (4)	0.74 (2)
H7A	0.3191	0.6916	0.8480	0.115*	0.74 (2)
H7B	0.2935	0.5229	0.7469	0.115*	0.74 (2)
C8	0.2826 (2)	0.392 (3)	0.9062 (8)	0.108 (3)	0.74 (2)
H8A	0.2596	0.5159	0.9088	0.162*	0.74 (2)
H8B	0.2983	0.3773	0.9821	0.162*	0.74 (2)
H8C	0.2726	0.2085	0.8809	0.162*	0.74 (2)
C7'	0.3026 (5)	0.427 (8)	0.800 (3)	0.090 (7)	0.26 (2)
H7'1	0.2834	0.2723	0.7812	0.109*	0.26 (2)
H7'2	0.2984	0.5626	0.7372	0.109*	0.26 (2)
C8'	0.2950 (11)	0.565 (9)	0.913 (2)	0.119 (8)	0.26 (2)
H8'1	0.2672	0.6353	0.9054	0.178*	0.26 (2)
H8'2	0.3139	0.7185	0.9309	0.178*	0.26 (2)
H8'3	0.2990	0.4293	0.9748	0.178*	0.26 (2)
N1	0.45462 (6)	−0.1686 (4)	0.80964 (16)	0.0371 (4)	
Cl1	0.530459 (19)	0.33620 (12)	0.89413 (4)	0.04150 (19)	
Zn1	0.5000	0.06363 (7)	0.7500	0.03228 (16)	
H1A	0.4494 (7)	−0.320 (4)	0.7676 (17)	0.048 (7)*	
H1B	0.4647 (7)	−0.216 (5)	0.8810 (14)	0.058 (8)*	

### Atomic displacement parameters (Å^2^)

**Table:**

	*U*^11^	*U*^22^	*U*^33^	*U*^12^	*U*^13^	*U*^23^
C1	0.0385 (13)	0.0332 (11)	0.0360 (12)	−0.0035 (11)	0.0103 (10)	0.0059 (9)
C2	0.0492 (15)	0.0410 (13)	0.0398 (12)	0.0043 (12)	0.0059 (10)	0.0019 (10)
C3	0.0674 (19)	0.0497 (16)	0.0624 (17)	0.0114 (16)	0.0253 (15)	0.0013 (13)
C4	0.0467 (16)	0.068 (2)	0.080 (2)	0.0142 (16)	0.0189 (15)	0.0256 (16)
C5	0.0452 (17)	0.104 (3)	0.0649 (19)	−0.0029 (18)	−0.0070 (14)	0.0154 (18)
C6	0.0479 (17)	0.0712 (18)	0.0472 (15)	−0.0081 (15)	0.0034 (13)	−0.0095 (13)
C7	0.061 (4)	0.103 (7)	0.129 (7)	0.030 (4)	0.036 (4)	0.032 (5)
C8	0.060 (4)	0.119 (7)	0.155 (6)	0.017 (4)	0.048 (4)	0.007 (5)
C7'	0.069 (10)	0.075 (11)	0.126 (11)	0.019 (8)	0.010 (9)	0.019 (9)
C8'	0.093 (14)	0.131 (16)	0.136 (13)	0.051 (12)	0.031 (11)	0.011 (13)
N1	0.0438 (12)	0.0288 (10)	0.0396 (11)	0.0005 (9)	0.0087 (9)	0.0011 (8)
Cl1	0.0565 (4)	0.0367 (3)	0.0300 (3)	0.0007 (3)	0.0015 (2)	−0.0050 (2)
Zn1	0.0388 (3)	0.0283 (2)	0.0307 (2)	0.000	0.00804 (16)	0.000

### Geometric parameters (Å, º)

**Table:**

C1—C6	1.367 (3)	C8—H8A	0.9600
C1—C2	1.372 (3)	C8—H8B	0.9600
C1—N1	1.441 (3)	C8—H8C	0.9600
C2—C3	1.377 (4)	C7'—C8'	1.527 (19)
C2—H2	0.9300	C7'—H7'1	0.9700
C3—C4	1.370 (4)	C7'—H7'2	0.9700
C3—H3	0.9300	C8'—H8'1	0.9600
C4—C5	1.377 (4)	C8'—H8'2	0.9600
C4—C7	1.521 (6)	C8'—H8'3	0.9600
C4—C7'	1.521 (10)	N1—Zn1	2.0478 (19)
C5—C6	1.381 (4)	N1—H1A	0.875 (16)
C5—H5	0.9300	N1—H1B	0.881 (16)
C6—H6	0.9300	Cl1—Zn1	2.2409 (5)
C7—C8	1.500 (11)	Zn1—N1^i^	2.0478 (19)
C7—H7A	0.9700	Zn1—Cl1^i^	2.2409 (6)
C7—H7B	0.9700		
			
C6—C1—C2	119.7 (2)	H8A—C8—H8B	109.5
C6—C1—N1	120.6 (2)	C7—C8—H8C	109.5
C2—C1—N1	119.6 (2)	H8A—C8—H8C	109.5
C1—C2—C3	119.8 (2)	H8B—C8—H8C	109.5
C1—C2—H2	120.1	C4—C7'—C8'	108.5 (16)
C3—C2—H2	120.1	C4—C7'—H7'1	110.0
C4—C3—C2	122.1 (3)	C8'—C7'—H7'1	110.0
C4—C3—H3	119.0	C4—C7'—H7'2	110.0
C2—C3—H3	119.0	C8'—C7'—H7'2	110.0
C3—C4—C5	116.8 (3)	H7'1—C7'—H7'2	108.4
C3—C4—C7	117.7 (7)	C7'—C8'—H8'1	109.5
C5—C4—C7	125.4 (7)	C7'—C8'—H8'2	109.5
C3—C4—C7'	133.8 (10)	H8'1—C8'—H8'2	109.5
C5—C4—C7'	108.9 (10)	C7'—C8'—H8'3	109.5
C7—C4—C7'	18.8 (14)	H8'1—C8'—H8'3	109.5
C4—C5—C6	122.3 (3)	H8'2—C8'—H8'3	109.5
C4—C5—H5	118.9	C1—N1—Zn1	112.09 (13)
C6—C5—H5	118.9	C1—N1—H1A	110.0 (16)
C1—C6—C5	119.3 (3)	Zn1—N1—H1A	110.4 (16)
C1—C6—H6	120.3	C1—N1—H1B	109.2 (17)
C5—C6—H6	120.3	Zn1—N1—H1B	105.3 (17)
C8—C7—C4	112.3 (8)	H1A—N1—H1B	110 (2)
C8—C7—H7A	109.1	N1^i^—Zn1—N1	114.80 (11)
C4—C7—H7A	109.1	N1^i^—Zn1—Cl1^i^	108.97 (6)
C8—C7—H7B	109.1	N1—Zn1—Cl1^i^	107.31 (6)
C4—C7—H7B	109.1	N1^i^—Zn1—Cl1	107.31 (6)
H7A—C7—H7B	107.9	N1—Zn1—Cl1	108.97 (6)
C7—C8—H8A	109.5	Cl1^i^—Zn1—Cl1	109.41 (3)
C7—C8—H8B	109.5		
			
C6—C1—C2—C3	1.5 (4)	C3—C4—C7—C8	−77.7 (15)
N1—C1—C2—C3	178.0 (2)	C5—C4—C7—C8	104.8 (14)
C1—C2—C3—C4	0.1 (4)	C7'—C4—C7—C8	74 (5)
C2—C3—C4—C5	−1.8 (4)	C3—C4—C7'—C8'	−4 (5)
C2—C3—C4—C7	−179.6 (5)	C5—C4—C7'—C8'	167 (3)
C2—C3—C4—C7'	168 (2)	C7—C4—C7'—C8'	−39 (4)
C3—C4—C5—C6	2.0 (5)	C6—C1—N1—Zn1	95.6 (2)
C7—C4—C5—C6	179.6 (6)	C2—C1—N1—Zn1	−80.9 (2)
C7'—C4—C5—C6	−170.4 (17)	C1—N1—Zn1—N1^i^	−159.46 (17)
C2—C1—C6—C5	−1.3 (4)	C1—N1—Zn1—Cl1^i^	−38.19 (16)
N1—C1—C6—C5	−177.8 (2)	C1—N1—Zn1—Cl1	80.18 (15)
C4—C5—C6—C1	−0.5 (5)		

Symmetry code: (i) −*x*+1, *y*, −*z*+3/2.

### Hydrogen-bond geometry (Å, º)

**Table:**

*D*—H···*A*	*D*—H	H···*A*	*D*···*A*	*D*—H···*A*
C2—H2···Cl1^ii^	0.93	2.94	3.630 (2)	132
N1—H1*A*···Cl1^iii^	0.88 (2)	2.65 (2)	3.424 (2)	149 (2)
N1—H1*B*···Cl1^iv^	0.88 (2)	2.66 (2)	3.5083 (19)	161 (2)

Symmetry codes: (ii) −*x*+1, −*y*+1, −*z*+2; (iii) −*x*+1, *y*−1, −*z*+3/2; (iv) −*x*+1, −*y*, −*z*+2.
