# Supplementary figures and images for: Crystal structure of di­chlorido­bis­(4-ethyl­aniline-κN)zinc
Source: Acta Crystallogr E Crystallogr Commun. 2015 Jan 14;71(Pt 2):m21–2. doi: 10.1107/S2056989014027832 (PMC4384566; doi:10.1107/S2056989014027832)

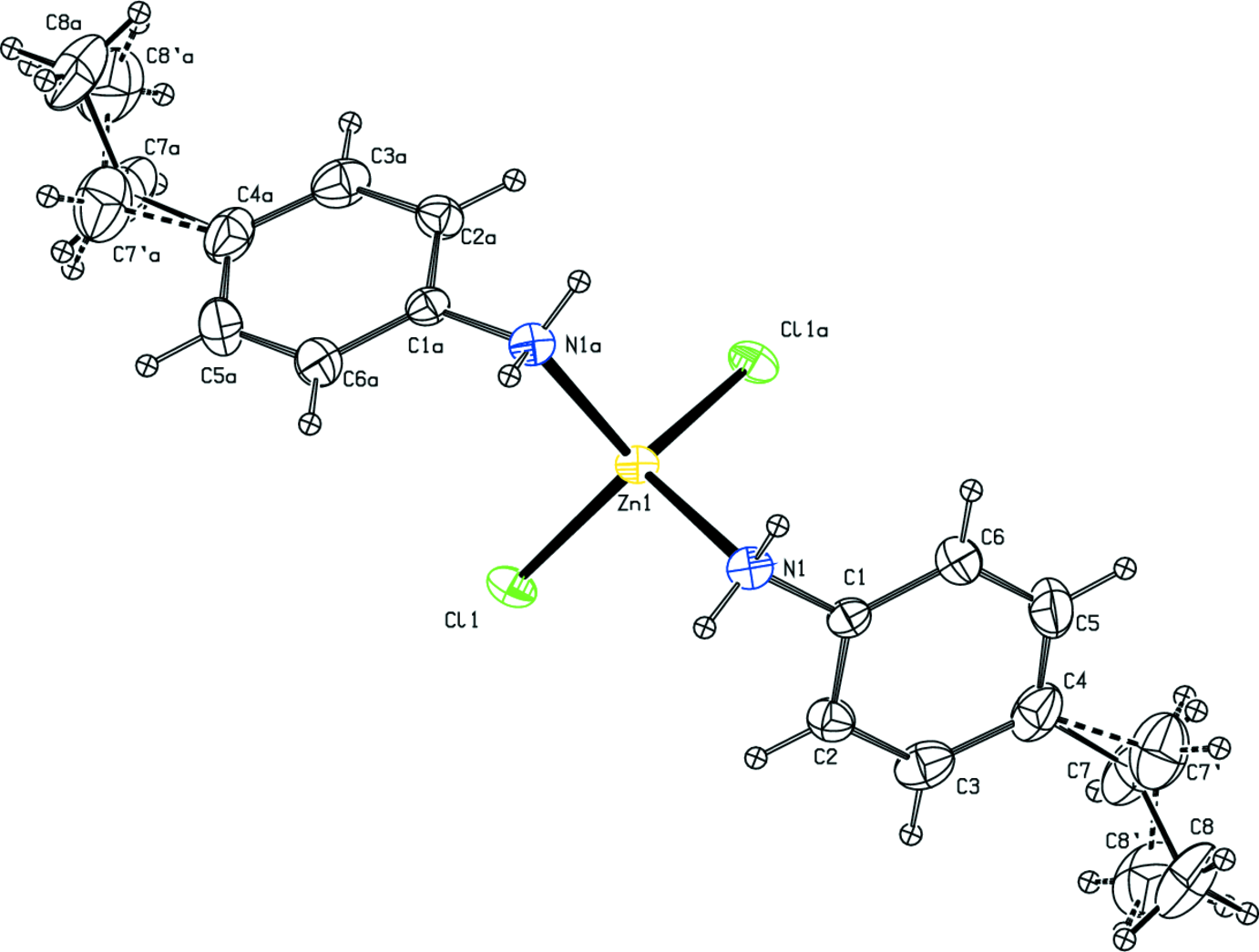

Supplement: Supplementary file 3 [file e-71-00m21-fig1.tif]

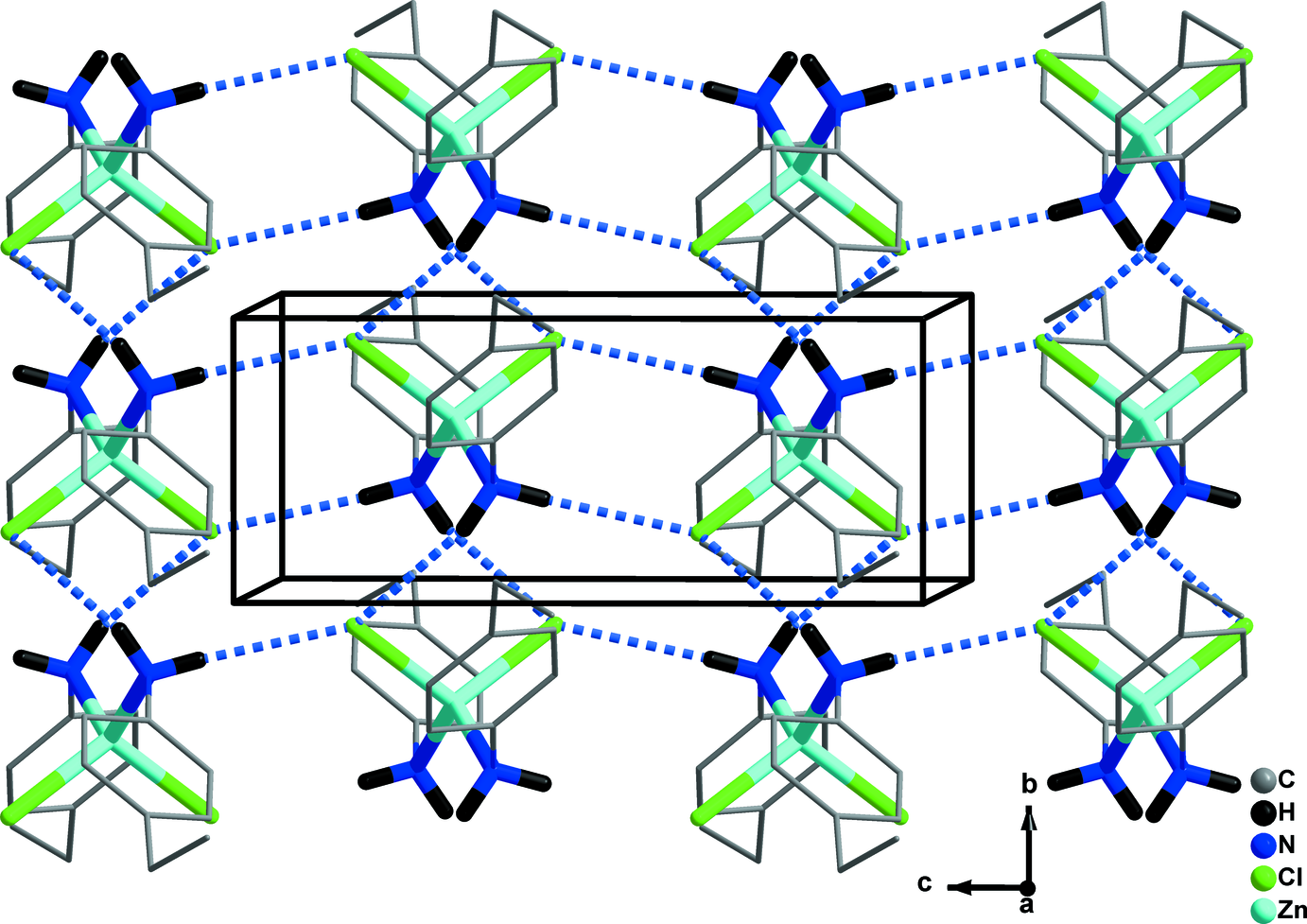

Supplement: Supplementary file 4 [file e-71-00m21-fig2.tif]
